# Effect of Laser Parameters on Surface Morphology and Material Removal Mechanism of Ablation Grooves in CFRP Composites Using Finite Element Simulations

**DOI:** 10.3390/ma18040790

**Published:** 2025-02-11

**Authors:** Juan Song, Bangfu Wang, Qingyang Jiang, Xiaohong Hao

**Affiliations:** 1 Department of Basic Courses, Suzhou City University, Suzhou 215104, China; 2 College of Mechanical Engineering, Suzhou University of Science and Technology, Suzhou 215009, China

**Keywords:** CFRP, laser ablation, surface morphology, heat-affected zone, finite element simulation, process parameters

## Abstract

Carbon fiber resin matrix composites (CFRP) are widely recognized for their exceptional properties such as high temperature resistance and high strength, making them indispensable in aerospace, automotive, and medical applications. Despite their growing use, precision machining of CFRP remains challenging. Traditional mechanical machining methods often lead to severe tool wear, matrix damage, fiber pullout, delamination, and chipping. In contrast, nanosecond pulsed laser machining has garnered significant attention due to its high precision, minimal heat-affected zone (HAZ), and versatility in processing various materials. In this study, a finite element model was developed to account for the anisotropic heat transfer and non-homogeneous properties of CFRP, enabling accurate simulation of laser machining processes. The study analyzed the influence of laser parameters on machining quality and revealed the ablation mechanism and HAZ evolution under varying laser conditions. Notably, it was observed that the thermal conductivity along the carbon fiber’s axial direction is higher than in the radial direction, resulting in an elliptical ablation pattern after laser irradiation. Additionally, the effects of the laser power, pulse frequency, and scanning speed on the depth and width of grooves were investigated through finite element simulations and validation experiments. A heat accumulation effect between laser pulses was observed, where resin matrix material around the grooves was removed once the accumulated heat exceeded the resin’s pyrolysis temperature. In addition, if there is too much laser power or too small a laser scanning speed, the fiber will undergo severe ablation removal, which will form serious thermal damage and a heat-affected zone. Gradually increasing the laser power or decreasing the scanning speed led to deeper and wider grooves, with an inverted triangular morphology. Moreover, the selection of different parameters had a significant effect on the ablation morphology, heat-affected zone, and the contour parameters of the grooves. This research contributes to understanding the laser–CFRP interaction mechanism and offers insights for optimizing laser processing parameters to improve material processing accuracy and efficiency, further expanding the potential applications of laser technology in composite material machining.

## 1. Introduction

Carbon fiber-reinforced plastic (CFRP) is a composite material with carbon fiber as the reinforcement and resin as the matrix [1,2]. It has excellent properties such as low density, high strength, high temperature resistance, radiation resistance, and chemical corrosion resistance [3,4], and is one of the most popular high-performance materials, which is widely used in aerospace, wind power generation, sporting goods, vehicles and rail transportation, and ocean engineering [5,6]. After molding of CFRP, a large number of small holes need to be processed in most cases to meet the requirements of assembly, linking, etc. The machining quality of connection holes is directly related to the quality of aircraft assembly and aircraft life [7,8]. Due to the different properties of the matrix and reinforcing phase, CFRP is an anisotropic and non-homogeneous material, the hardness of carbon fiber is very high, and there are great difficulties in its secondary processing [9,10].

At present, there are two main categories of a hole-making process for CFRP; one is a traditional machining process, such as milling and drilling [11]. The other category is special machining processes, such as electric discharge machining (EDM), laser machining, ultrasonic drilling, water jet machining, and their composite machining techniques [12,13,14]. Although traditional machining methods are characterized by high efficiency, CFRP plates processed by such methods suffer from fiber pullout, lack of surface integrity, and tool wear [15,16]. Several non-traditional machining methods have been proposed to process difficult materials, significantly improving the mechanical and chemical properties of the components [17]. CFRP samples were machined by abrasive water jet machining without a heat-affected zone (HAZ), but there was carbon fiber pullout and delamination due to the impact of water jets. Laser-induced plasma micromachining (LIPMM) has a small HAZ, little thermal damage, and is used to drill micro-holes in CFRP with a small HAZ and excellent roundness [18]. However, the LIPMM process is only suitable for machining micro- and nanostructures. EDM is an ideal method for machining CFRP with a smooth cross-section, but frequent electrode changes can limit the efficiency of EDM [19].

In recent years, laser processing technology has been developing rapidly, which has the significant advantages of high precision and high efficiency [20,21], and has an obvious enhancement effect on the shape accuracy of the processed material [22], and its application potential in the field of composite processing as a non-contact advanced processing technology is huge [23], so laser processing of CFRP has become a hot spot of research nowadays [24]. V. Oliveira et al. [25] investigated the use of femtosecond laser ablation to process the preparation of material bonding surfaces and evaluated the potential of this method to improve the bond strength of carbon fiber composites. When the processing parameters were chosen such that the laser intensity was above the removal threshold for the resin matrix and below that for the carbon fibers, the resin matrix on the surface of the CFRP could be selectively removed, exposing the carbon fibers inside, and periodic ripples were found on the surface of the carbon fibers [26]. Kumar et al. [27] achieved the selective processing of low-spatial-frequency microstructures and high-spatial-frequency microstructures on CFRP using a femtosecond laser, and the results showed that parameters such as the pulse energy, amount of defocusing, and other parameters have an effect on the wettability of the periodic ripples as well as the spatial period. Leone et al. [28] investigated by a quasi-continuous-wave fiber laser that the use of high pulse power (up to 4.5 kW) and short pulse duration (0.05 ms) during processing of CFRP can reduce HAZ formation and achieve high cutting speeds. A narrow kerf (less than 200 μm) and limited HAZ (about 0.5 mm) can be obtained by the proper selection of the process parameters. In addition, the heat-affected zone measured on the bottom surface can be effectively used as a damage indicator to understand the overall thermal damage. Ohkubo et al. [29] analyzed the effect of the difference in the ablation rate between carbon fiber and epoxy resin substrates on the machining quality by simulation. Due to the faster thermal conductivity of the carbon fibers, the corrosion rate of the carbon fibers is faster than that of the epoxy resin in the first stage, and based on numerical simulation, the mechanism of laser material removal was revealed, and it was found that the surface quality of laser-processed CFRP was lower due to the different material removal rates of the fiber and matrix. Subsequently, Contuzzi et al. [30] used finite element simulation to analyze the effect of the process parameters of laser ablation on the depth of ablation and surface quality of CFRP plates and found that laser processing can improve the surface quality and processing efficiency of CFRP by optimizing the laser processing parameters and revealing the mechanism of material removal and the mechanism of thermal damage formation. Ohkubo et al. [31] applied the finite difference method to the numerical simulation of material removal in CFRP laser ablation, and it is important to consider the combustion effect when discussing the generation of an HAZ.

Due to the great differences between the thermodynamic properties of carbon fibers in CFRP [32], such as the coefficient of thermal expansion, vaporization temperature, and thermal conductivity, and the resin matrix, defects such as the HAZ, fiber pullout, delamination of composites, and fiber-end expansion are prone to occur during laser processing [33,34]. Although the optimal process parameters during laser processing can be obtained experimentally, it is time-consuming and expensive to conduct response surface or one-factor experiments [35]. In addition, the laser parameters obtained through process parameter optimization experiments are generally only suitable for one material or one working condition and may not be suitable for other materials or other working conditions [36]. Therefore, many researchers have used finite element simulation to analyze the material removal mechanism and process parameter optimization during laser processing [37,38,39]. Wang et al. [15] developed a novel numerical model considering anisotropic heat transfer to analyze the heat transfer and thermal damage of CFRP laser processing. Deeper and narrower microgrooves were obtained when the laser scanning direction was parallel to the carbon fiber direction, and the laser processing resulted in severe heat buildup at the edges of the CFRP, and the effects of parameters such as the laser energy, scanning speed, and scanning space on the depth and width of the laser ablation were also explored. Further, Wang et al. [40] investigated the ablation mechanism of laser-processed CFRP under different scanning directions by comparing finite element simulations with validation experiments under different scanning directions. It was found that the width of the HAZ and ablation increased with the scanning angle and decreased with the scanning speed. Secondly, the researchers attempted to remove epoxy and contaminants from CFRP using a nanosecond laser, and investigated the effects of temperature field distribution, laser power density, and laser scanning speed on the cleaning effect to provide a theoretical reference for the cleaning experiments. It was found that the cleaned surfaces exhibited higher surface free energy, which resulted in improved tensile shear strength and flexural strength [41]. Although a number of studies have provided some insights into the optimization of laser parameters in a number of aspects, the mapping of laser parameters to the processed surface morphology and material removal mechanisms has not yet been fully and explicitly elaborated, especially the comprehensive analysis of process parameters in the composite removal process.

In this paper, in order to accurately describe the anisotropic heat transfer, material removal mechanism, and thermal damage formation of CFRP processed by a nanosecond laser, a finite element model that takes into account the anisotropic heat transfer and inhomogeneity of the material is established. In addition, the influence law of the laser parameters on the processing quality was analyzed, and the ablation mechanism and the evolution of an HAZ under different laser parameters were revealed by combining numerical simulations. Meanwhile, a validation experiment of the laser single scratching of CFRP was carried out to analyze the effects of the laser power and laser scanning speed on the width and depth of the groove, which provides technical support for the subsequent high-quality and efficient processing of CFRP.

## 2. Materials and Methods 

### 2.1. Modeling and Simulation of Laser-Ablated CFRP

#### 2.1.1. Analysis of Ablation Behavior of CFRP

The materials used in this study are CFRP, in which the fibers are carbon fibers, and the carbon fiber prepreg is processed by coating, hot pressing, cooling, and laminating, which is a thermoplastic resin. When the laser temperature exceeds 300 °C, the fiber undergoes an oxidaion reaction, and different oxidation products are produced at different temperatures; usually, carbon monoxide and carbon dioxide gases are produced after the fiber oxidation. In addition, the matrix of CFRP is a resin, which first decomposes when heated and subsequently vaporizes at transient high temperatures. The products are all gaseous substances.

#### 2.1.2. Finite Element Modeling

**Figure 1:**
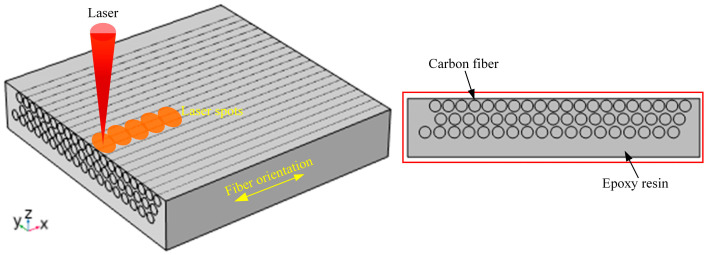
Finite element model.

CFRP is a typical anisotropic, non-homogeneous difficult-to-process composite material; the laser action on the CFRP needs to take into account the anisotropy of the material. This paper uses COMSOL Multiphysics software (v.6.1) to establish a finite element model of the laser ablation temperature field of CFRP, as shown in Figure 1. COMSOL’s graphical user interface was utilized for the geometric construction of the model, the definition of material properties, and the setting of boundary conditions. The model considers the anisotropic heat transfer of CFRP, coupled with solid heat transfer and deformation, which can provide theoretical guidance for the ablation depth and heat-affected zone of CFRP for laser processing. In addition, Section 2.1.3, Section 2.1.4, and Section 2.1.5 provide theoretical formulas for the equations of the heat source and heat flux in the model.

The size of CFRP in the finite element model is 100 × 100 × 20 μm, the diameter of the fibers is 4 μm and the length is 100 μm, and the fibers are uniformly distributed in the interior of the matrix, which is a resin. The transient study is used in this model, the material type is solid, and the heat source is a generalized source. Then, the material properties of the resin are mainly considered parameters such as the density, thermal conductivity, and heat capacity. The material properties of the fiber are mainly in the thermal conductivity, which needs to be considered in the axial direction of the fiber and the radial direction of the fiber; this is because of the anisotropy of the fiber. The temperature is discretized using quadratic Lagrangian units. In addition, the initial values are chosen as the heat source and solid, and the temperature value is set to 293.15 K. The heat flux is used as convective heat flux and the heat transfer coefficient is set to 10 and the material type is non-solid. And the specific parameters of the material are shown in Table 1.

A nanosecond pulsed laser in the ablation of CFRP involves the extension of the ablation surface of the target material, the XY plane, and the advancement of the ablation depth in the Z direction. Since the pulse width of a nanosecond pulsed laser is very small (nanosecond scale), the macroscopic distance (the ablation depth) over which thermal energy can be conducted is very small. The diffusion distance of laser energy over the ablated surface is in the order of millimeters, and the difference between the two is large. Relative to the ablation depth of the laser-processed material, the diffusion and conduction distance of thermal energy on the ablation surface can be regarded as infinite, so the three-dimensional thermal energy conduction problem of the pulsed laser processing of CFRP can be reduced to a one-dimensional problem to be dealt with, with the laser beam directed vertically to the surface of the CFRP, and with the thermal energy conduction along the radial target material.

#### 2.1.3. Coupled Physical Modeling of Laser Ablation

During the processing of laser CFRP panels, the laser beam has a certain moving speed, so the heat source needs to be calibrated. In this paper, a line Gaussian heat source is proposed to simulate the fast-moving temperature field of laser ablation. Setting the laser line scanning speed as *v*, the laser scanning length as *L*, the ablation time as *t* = *L*/*v*, and the total heat input during the ablation process as *Q*, Equation (1) is obtained [47].

(1) Q=Pt=LP/1000v

where *Q* is the total heat input during the ablation process, J; *P* is the laser power, W; *L* is the laser scanning length, μm; *v* is the scanning speed, mm/s.

The distribution of material femtosecond laser energy is usually Gaussian and can be replaced by a Gaussian heat source when simulation is performed. The absorption rate of laser energy is different for different materials, so the energy absorption rate needs to be taken into account when using the equivalent heat flux. In laser processing, the laser spot is irradiated on the surface of the material, and the laser energy is mainly absorbed by the surface of the material, so the process of transferring energy from the laser beam to the surface of the material can be simplified as the loading of a surface heat source. The laser satisfies the Gaussian distribution, as shown in Equation (2) [48].

(2) $$q(t)=q_{m}\exp[-\frac{{\left(t-0.5\tau \right)}^{2}}{2\sigma^{2}}]$$

where *q_m_* is the heat flow density in the center region of the spot, W/μm^2^; *τ* is the laser pulse width, ns; *σ* is a parameter that can change the shape of the laser pulse time; *t* is the ablation time, ns. The heat source model has equal thermal energy input in both cases, and the joint Equations (3) and (4) are obtained as follows:

(3) $$2\int_{0}^{L}\int_{0}^{\infty }q_{m}\exp[-\frac{{\left(y-0.5\tau \right)}^{2}}{2\sigma^{2}}]=LP/v$$

(4) $$q(t)=\frac{P}{\sqrt{2\pi }\sigma v}\exp[-\frac{{\left(t-0.5\tau \right)}^{2}}{2\sigma^{2}}]$$

where *y* is the Y-axis coordinate of the spot, μm.

In order to better describe the distribution law of the intensity of the laser energy, the laser power density *I*(*x*,*y*) is obtained by considering the beam shape of the laser as well as the absorption at different light source positions, as shown in Equations (5) and (6).

(5) $$I(x,y)=AI_{0}e^{\frac{{\left(x-x_{0}\right)}^{2}+{\left(y-y_{0}\right)}^{2}}{r^{2}}}$$

(6) $$I_{0}=\frac{2P}{\tau \pi r^{2}\left(1-e^{2}\right)}$$

where *A* is the energy absorption rate; *x*_0_ is the horizontal coordinate of the center of the laser source, μm; *y*_0_ is the vertical coordinate of the center of the laser source, μm; *r* denotes the radius of the laser focal point, μm; *x* is the distance between the incident laser and the surface of the CFRP sheet, μm; and *q*(*t*) is the Gaussian heat source.

The initial temperature was set to room temperature (about 300 K), and the conduction of thermal energy at the surface of the carbon fiber material is the absorption of laser energy by the surface of the material, with no further transfer of thermal energy at the maximum ablation depth of the material. The following boundary conditions were established:

(7) $$T(x,t)=T_{0},t=0$$

(8) $$-k{\left.\frac{\partial T}{\partial x}\right|}_{x=0}=b\frac{P}{\sqrt{2\pi }}\exp(-\frac{\tau^{2}}{8\sigma^{2}}),0\leq t\leq \tau$$

(9) $$-k{\left.\frac{\partial T}{\partial x}\right|}_{x=\delta }=0,0\leq t\leq \tau$$

where *T* is the material surface temperature, *K*; *b* is the absorption of the laser by the CFRP, μm; *k* is the heat diffusion coefficient.

#### 2.1.4. Meshing and Boundary Conditions

The thermal conductivity along the carbon fiber axis is much larger than that along the radial direction. Therefore, the anisotropic heat transfer cannot be ignored or simplified in the numerical analysis of laser-processed CFRP, improving the simulation accuracy of laser ablation in CFRP. The material at the bottom of the workpiece is set as a thermally insulating boundary condition. In this study, the thermal conductivity tensor with three different components is applied to describe the anisotropic heat transfer in carbon fibers, denoted as Equation (10).

(10) $$k=\left[\begin{array}{c} K_{xx}\;K_{xy}\;K_{xz}\\ K_{yx}\;K_{yy}\;K_{yz}\\ K_{zx}\;K_{zy}\;K_{zz} \end{array}\right]=\left[\begin{array}{c} 50\;0\;0\\ 0\;5\;0\\ 0\;0\;5 \end{array}\right]$$

where *K_xx_*, *K_yy_*, and *K_zz_* are thermal conductivities in the *x*, *y*, and *z* directions, respectively.

Secondly, the heat transfer during nanosecond laser processing of CFRP satisfies the energy conservation theorem and Fourier’s law of heat transfer, as shown in Equations (11) and (12).

(11) $$-\frac{\partial q}{\partial n}+Q=PC\frac{\partial T}{\partial n}$$

(12) $$q=-K(T)\frac{\partial T}{\partial n}$$

The mesh was carefully constructed based on the physical problem under study and the geometric model features. During the meshing process, mesh refinement was carried out and local encryption operations were performed mainly at the boundary layers and material surfaces. The shape of the mesh is a free triangle, and the maximum cell of the mesh is 8, the minimum cell is 1, and the curvature factor is 0.5. In addition, the initial time step is set to 3.36 × 10^−9^ s. In the simulation process, an adaptive time-step strategy is adopted to ensure the stability and accuracy of the calculation. After the initial time step, the time step can be appropriately increased for the second time step of 1.68 × 10^−6^ s. Secondly, the simulation calculations are performed on a 12th Gen Intel^®^ Core^TM^ i5-12500H processor (Intel Corporation, Santa Clara, CA, USA) with 16 GB of RAM (Kingston, Shenzhen, China). The nanosecond laser finite element simulation was set up as a one-factor experiment to analyze the effects of the laser power, laser pulse frequency, and laser scanning speed on the ablation morphology and temperature field of CFRP, respectively, and the specific simulation parameters are displayed in Table 2.

It should be noted that the parameters of the laser device in Table 2 are sourced from the manufacturer. Secondly, the power, pulse frequency, and scanning speed of the laser are selected after pre-testing based on the processing parameters in the actual production process of CFRP, and the horizontal spacing of the laser parameters is the same.

#### 2.1.5. Control Equations and Assumptions

The material removal simulation process is roughly as follows: the equivalent heat flux of the laser is loaded on the surface of the finite element model, the material absorbs the energy and the temperature rises rapidly, reaching the melting gasification temperature of the material, the material is removed, and an ablated morphology is formed. The residual heat will continue to require heat conduction within the material, creating an HAZ. The role of the laser and the material process is very complex, also affecting the processing results of the factors very much, such as the material’s own physical specificity, laser processing parameters, and processing environment. Therefore, it is necessary to perform some simplification of the simulation process; the simulation of the assumptions are as follows:

1. The nanosecond laser energy beam becomes a first-order Gaussian distribution and will be replaced with a Gaussian heat source;
2. The material is completely removed once it reaches the vaporization temperature of the component material;
3. Heat generated within the material due to chemical reactions occurring during processing is not considered;
4. Heat loss from thermal radiation is not considered and the laser energy is fully absorbed by the material;
5. The absorption of nanosecond laser energy by the material is constant;
6. The focal plane is assumed to be the processing plane and the spot diameter does not change;
7. The resin matrix is heated and decomposes directly into the residual carbon and organic gases of the final product, without regard to multi-step pyrolysis reactions and intermediate pyrolysis products of the material;
8. The volume change in the composite material during the body ablation process is neglected.

In addition, the boundary condition of the laser processing region is the convective heat transfer between the laser heat source and the surrounding medium. The additional note needs to be added as follows: (1) The material property approximation, in which the thermophysical properties such as thermal conductivity, specific heat capacity, and heat diffusion coefficient of the resin as well as the fibers in CFRP are considered as constants in the model (as shown in Table 1). (2) The geometric structure approximation. In this paper, the fiber distribution in CFRP and the macroscopic geometric structure of the material are simplified, for example, the random distribution of the fibers, the weaving method, and the inhomogeneity of the resin are ignored. (3) The laser energy distribution approximation. When the laser acts on CFRP, its energy distribution in space is not absolutely uniform. In the modeling process, this paper assumes that the laser energy is Gaussian-distributed and given specific parameters such as spot size and power.

### 2.2. Experimental Setup

**Figure 2:**
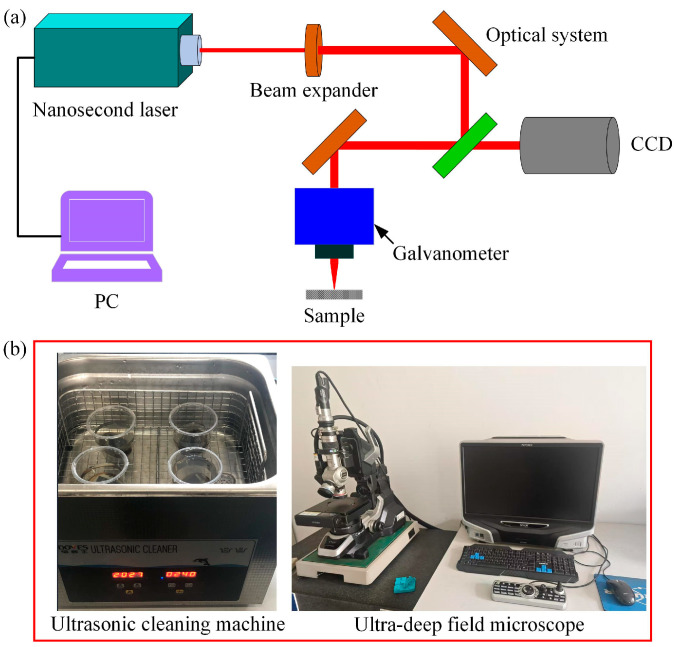
Schematic of nanosecond laser experimental system (**a**) and characterization method (**b**).

**Figure 3:**
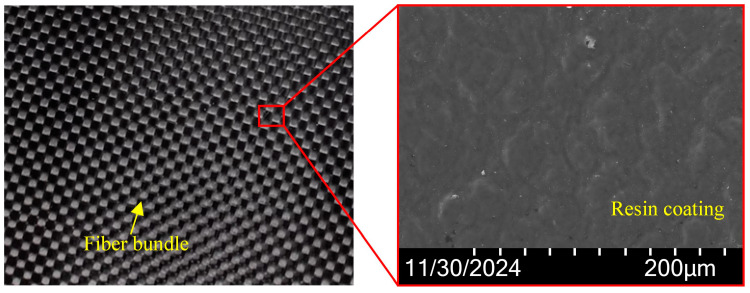
Initial morphology and elemental distribution of CFRP surface.

The experimental setup and characterization method of nanosecond laser ablation of CFRP grooves are shown in Figure 2. As shown in Figure 2a, the experimental system mainly consists of a nanosecond laser, beam expander mirror, reflector, focusing lens, three-axis carrier stage, and control system. In this experiment, the nanosecond laser device (BWT, Microtreat, Suzhou, China) was used to carry out the groove experiment under different laser parameters to investigate the influence law of the laser parameters on the groove morphology and contour. The specimen (as seen in Figure 3) was placed on the table, the laser spot was focused on the surface of the specimen, and the scanning was all performed with a single-direction motion, and the surface of the specimen was ultrasonically cleaned at the end of the experiment (ultrasonic cleaning machine, KQ-700V, Shumei, Shenzhen, China). In addition, as shown in Figure 10b, after cleaning and drying, the specimens were observed by an ultra-depth of field microscope (VHX6000, Keyence, Osaka, Japan) for the surface micromorphology and groove profile. Then, the microscopic morphology of the groove surface was tested by scanning electron microscopy (SEM, SU5000, Keyence, Japan). As a note, the depth of the groove is the distance from the surface of the material to the bottom of the groove, and the width is the width of the top of the groove. Each set of parameters was measured three times and averaged.

## 3. Results and Discussion

### 3.1. Evolution of the Transient Temperature Field Under Laser Action

**Figure 4:**
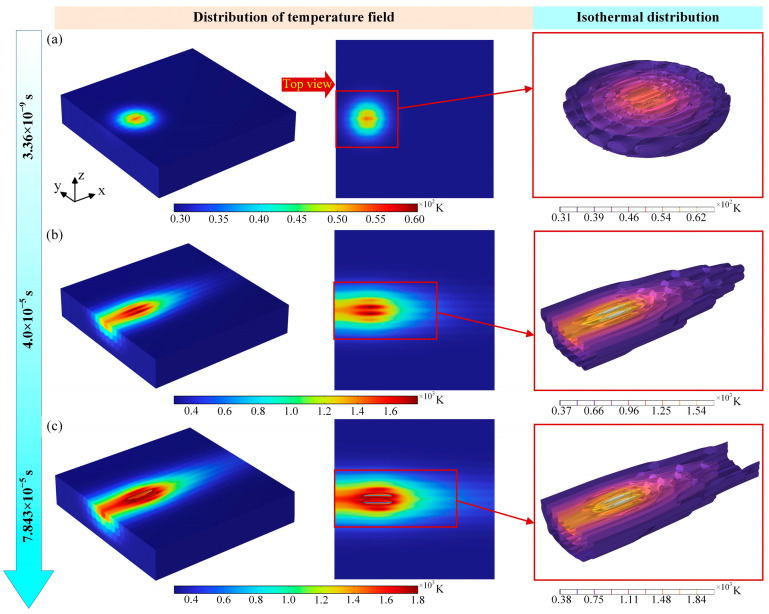
Evolution of transient temperature field with time during nanosecond pulsed laser ablation process: (**a**) *t* = 3.36 × 10^−9^ s, (**b**) *t* = 4.0 × 10^−5^ s, and (**c**) *t* = 7.843 × 10^−5^ s.

The ablation temperature field of the nanosecond pulsed laser at different laser parameters was calculated separately in the software. Figure 4 displays the transient temperature field of nanosecond pulsed laser-induced ablation at different times. In the pulsed laser mode, the nano-laser is irradiated on the surface of the CFRP material in 3.36 × 10^−9^ s time, and the high temperature is rapidly generated in a very short time, and the laser realizes the purpose of ablating the workpiece material. Secondly, as shown by the isotherms in Figure 4a, in the process of nanosecond laser action, on the one hand, it is the pulse width time during which the laser continuously applies thermal energy to the surface of the material. On the other hand, it is the non-pulse width time during which the laser energy is not input to the surface of the material, resulting in the attenuation of the laser energy on the surface of the material, which cannot be effectively ablated and removed, and the formation of pits with unstable ablation depth. Further, increasing the time of laser action on the material surface, as indicated in Figure 4b, when the laser power, scanning speed, and pulse frequency are fixed, the longer the laser action time, the higher the heat accumulated on the material surface, and the absorption of infrared laser light by carbon fibers accounted for about 80% of the laser light absorption, and the carbon fibers absorbed most of the laser light energy. Carbon fiber plays a dominant role in heat transfer in CFRP. In this case, the resin is first removed by carbon fiber heating, and then the fiber will begin to pyrolyze. In addition to that, when the laser action time continues to increase, the temperature of the material surface can obtain a maximum of 1800 K, which exceeds the decomposition temperature (698 K) and vaporization temperature (800 K) of the resin matrix. Pyrolysis and chemical reactions occur in large quantities under these conditions, affecting the laser processing results. The ablated carbon fibers will be oxidized first, and then as the temperature rises, carbon dioxide and carbon monoxide are generated from the complete and incomplete combustion of carbon material with oxygen, respectively. According to Figure 4c, it can be obtained that the fibers have undergone severe ablation removal, the laser energy of the laser spot continues to act, the heat quickly removes the carbon fibers, and at this time, it is easy for the ablation temperature to remove the epoxy resin, so there are a lot of carbon fibers exposed, which will form a serious thermal damage and heat-affected zone. At the same time, it can also be seen from Figure 2 that the depth of the ablation and HAZ of the laser is different at different times, and the overall presentation of the ablation morphology of the nanosecond pulsed laser is transformed from a circular to an elliptical shape with the increase in time, and the highest temperature occurs in the center region of the laser spot.

**Figure 5:**
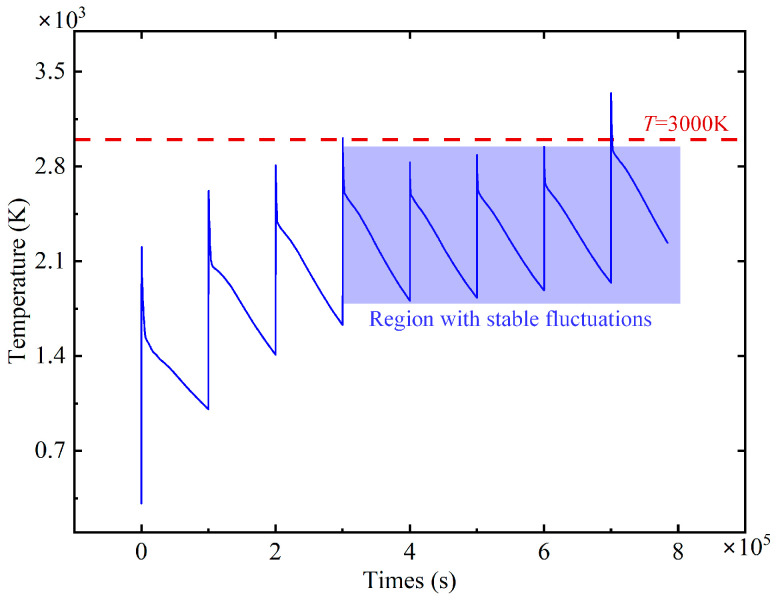
Temperature variation curve during laser ablation process.

According to the temperature field simulation results in Figure 5, the temperature profile shows relatively stable fluctuations near T = 3000 K. The temperature fluctuations in this region are small, and this region is defined as the stable fluctuation region. This region indicates that at a certain stage of laser ablation, the temperature is kept at a relatively constant level, but there are still small fluctuations. In addition, when the laser action time is within 3 × 10^−5^ s, the temperature profile shows larger fluctuations. The magnitude of temperature fluctuations in this region is significantly larger than that in the stable fluctuation region, which can be defined as the drastic fluctuation region. This region shows that in another stage of laser ablation, the temperature undergoes significant changes, the laser heating rate is relatively large, and the temperature is not higher than 2800 K. At the same time, the laser heat accumulated on the surface of the material is unstable, and the depth of ablation is about 10 μm.

### 3.2. Effect of Laser Power on Temperature Field and HAZ

**Figure 6:**
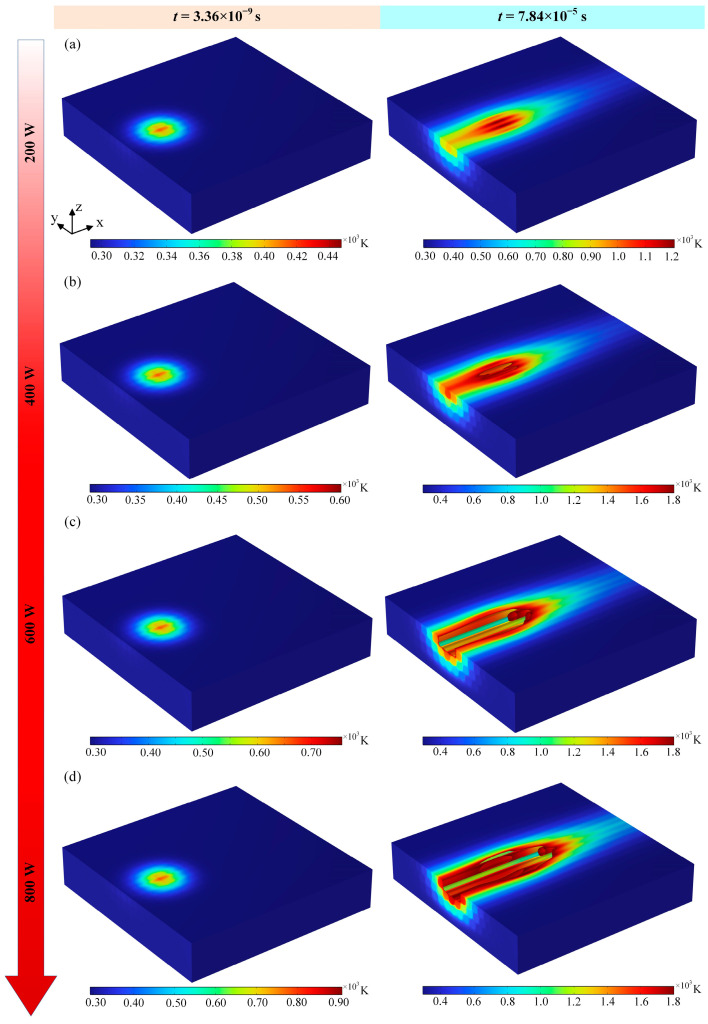
Temperature field clouds at different laser powers: (**a**) 200 W, (**b**) 400 W, (**c**) 600 W, and (**d**) 800 W.

As can be seen in Figure 6, the temperature field distribution on both the upper and lower surfaces of the material changes as the laser power changes. The laser power increases, the maximum temperature of the material surface increases, and the corresponding temperature field range also increases. When other conditions do not change, the laser power increases, the laser energy density on the surface of the material increases, the energy absorbed by the material increases, the thermal conductivity of the axial direction of the carbon fiber is large, and more heat will be rapidly transferred along the axial direction of the carbon fiber to the two sides of the slit, which leads to an increase in the width of the temperature field. When the laser power is 200 W, the temperature field of the surface of the material just contacted the spot range of the laser overall, which shows high in the middle and low at the edges, and when the laser action time is lengthened, the fiber ablation is more serious; Figure 6a–d all show the temperature distribution of the material under laser irradiation for a long period of time, and the highest temperature is located in the center. In addition, the energy of the nanosecond pulsed laser is Gaussian-distributed, and the power density in the middle region of the spot is higher, so the temperature is higher, causing the fiber to undergo ablation. Secondly, when increasing the laser power to 400 W, the temperature of the material in the center region of the laser irradiation is higher than the surrounding area, and the laser absorbs energy and passes through the carbon fiber radial and resin in the process of transferring energy from the top to the bottom, and the thermal conductivity of the resin and the carbon fiber radial is smaller. Compared to the carbon fiber axial direction, the heat transfer of the resin and carbon fiber radial direction is slow, and the fiber undergoes ablation removal, but the fiber at the edge of the center of the laser irradiation is not ablated. When the laser power is increased to 600 W and 800 W, as shown in Figure 6c,d, the evaporation temperature of the epoxy resin matrix (800 K) is lower than that of the carbon fibers (3900 K), which leads to the removal of the epoxy resin at a faster rate than that of the carbon fibers and the removal of the fibers by severe ablation, forming a long crater, with some fibers inside the material being exposed, and the epoxy resin around the fibers being removed by ablation.

**Figure 7:**
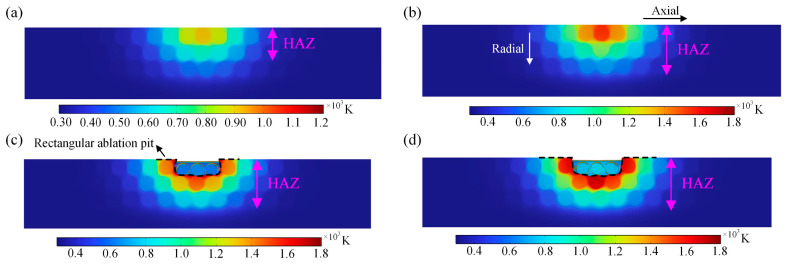
HAZ of cross-sections at different laser powers: (**a**) 200 W, (**b**) 400 W, (**c**) 600 W, and (**d**) 800 W.

Comparison of the variation in the HAZ in the cross-section of the material under different laser powers is shown in Figure 7. When the laser power is 200 W, the temperature in the center of the spot is relatively large, and the HAZ in the cross-section shows a semi-elliptic shape as a whole, which means that the temperature field directly affects the formation of the HAZ in the study of laser processing of CFRP. In addition, according to Figure 7b, the laser power is increased to 400 W, the temperature in the center region of the spot further increases, and the heat-affected zone further increases, but the energy transferred along the fiber radial direction is lower than that in the fiber axial direction, so that there is little change in the heat-affected zone and ablation pattern along the fiber radial direction. Further, the laser power is increased to 600 W. The epoxy matrix around the fibers is removed by ablation, and some of the fibers are also removed by ablation; finally, a rectangular ablation pit is formed, and the depth of the removed pit is about 4 μm (e.g., Figure 7c). In addition, due to the inconsistency of the thermal conductivity between the axial and radial directions of the fibers, the laser heat transfer rate in the axial direction of the fibers is relatively large, as shown in Figure 7d. The overall performance of the HAZ after laser irradiation is elliptical, and the energy of laser heat transfer to the lower surface of the material tends to increase. At the same time, the heat convection on the lower surface of the material is also lower than that on the upper surface, resulting in a larger range of heat-affected zones in the cross-section of the material.

### 3.3. Effect of Laser Pulse Frequency on Temperature Field and HAZ

**Figure 8:**
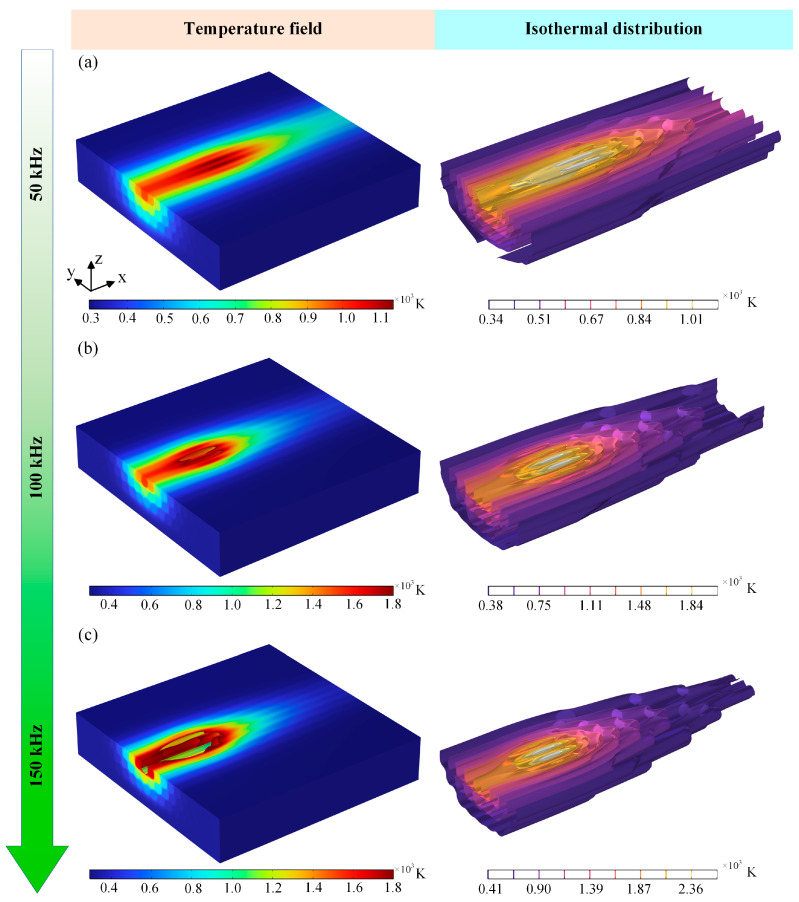
Temperature field clouds at different laser pulse frequencies: (**a**) 50 kHz, (**b**) 100 kHz, and (**c**) 150 kHz.

In order to investigate the ablation behavior and thermal effects of the laser pulse frequency on laser-machined CFRP, the power of the nanosecond pulsed laser was set to 400 W and the laser scanning speed was 130 mm/s. Single-cutting simulations were carried out using laser pulse frequencies of 50 kHz, 100 kHz, and 150 kHz, respectively. Figure 8 shows the temperature field cloud and isotherm distribution of CFRP cut with different laser pulse frequencies. From Figure 8a,b, it can be seen that the maximum temperature on the upper surface of the material increases with the increase in the laser pulse frequency, and the fibers in the center region of the laser spot are removed by ablation, which is relatively large at the laser center point. Secondly, from the isotherm distribution cloud diagram, it can also be found that after the increase in the laser pulse frequency, the length of the temperature field on the material surface parallel to the laser scanning path after laser irradiation decreases, but the width of the temperature field perpendicular to the direction of the laser scanning path increases. When the laser pulse frequency reaches 150 kHz, the ablation of the fiber at the center of the laser irradiation is more serious, and the ablation crater has an overall elliptical shape, as shown in Figure 8c. On the one hand, the increase in the number of laser pulses per unit time leads to an increase in the cumulative energy absorbed by the material, which in turn improves the ablation efficiency. This increase in energy density exacerbates the absorption of laser energy by the material, resulting in a more rapid and intense temperature increase on the upper surface of the material, melting and then vaporizing the resin, which leads to the formation of more significant ablation craters. On the other hand, the increase in the laser pulse frequency from 50 kHz to 150 kHz increases the temperature gradient on the material surface, intensifying the thermal stress effect, and the ablation crater gradually expands and takes on an irregular shape. Therefore, in the process of changing the laser pulse frequency from low to high, the degree of ablation on the surface of the CFRP material gradually increases, which further affects the complexity and irregularity of the ablation morphology, and the subsequent selection of the appropriate laser pulse frequency is of great significance for the control and optimization of the ablation morphology.

**Figure 9:**
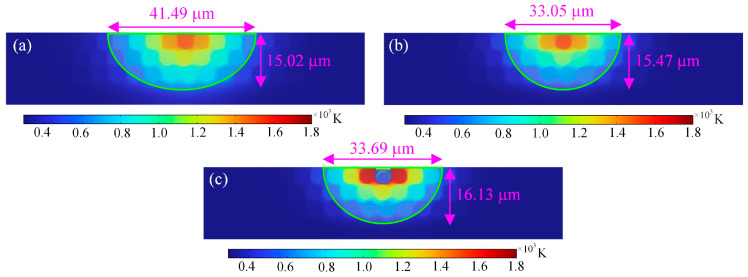
HAZ of cross-sections at different laser pulse frequencies: (**a**) 50 kHz, (**b**) 100 kHz, and (**c**) 150 kHz.

In order to quantify the effects of different laser pulse frequencies on the heat-affected zone of the ablated cross-section of CFRP, the evaluation indexes of the width and depth of the heat-affected zone of the cross-section of the material are proposed. Based on Figure 9, it can be found that as the laser pulse frequency increases from 50 kHz to 150 kHz, the depth of the heat-affected zone in the cross-section of the ablated material gradually increases, and the maximum can reach 16.13 μm, but the width of the heat-affected zone shows an overall trend of decreasing and then increasing, and the minimum width of the heat-affected zone is about 33.05 μm. When the laser pulse frequency increases, as illustrated in Figure 9c, some of the fibers in the material are removed by the ablation of the larger laser energy, but there are still some fibers in contact with oxygen and oxidation reaction occurs, and the fibers are vaporized and decomposed after absorbing the laser energy. The fibers in the CFRP material far away from the surface of the laser irradiation do not come into contact with the oxygen and have not yet reached the vaporization temperature of the fibers; therefore, the overall morphology of the fibers has not been changed.

### 3.4. Effect of Laser Scanning Speed on Temperature Field and HAZ

**Figure 10:**
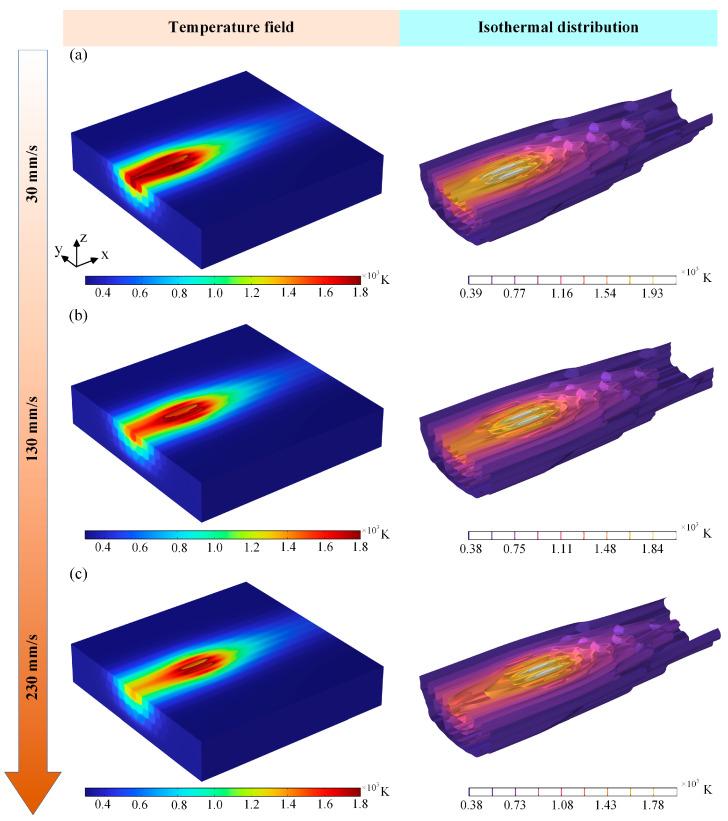
Temperature field clouds at different laser scanning speeds: (**a**) 30 mm/s, (**b**) 130 mm/s, and (**c**) 230 mm/s.

In order to investigate the variation in the laser scanning speed on the ablation behavior and thermal influence of laser-cut CFRP, the temperature field cloud plots at different laser scanning speeds are shown in Figure 10. As the laser scanning speed increases from 30 mm/s to 230 mm/s, the heat-affected zone of laser action gradually decreases, and the low laser scanning speed can cause more resin epoxy degradation and form extensive thermal damage. In addition, due to the poor thermal convection between the material and air, severe heat can accumulate at the edge of the material during laser processing, which in turn leads to severe thermal damage. According to Figure 10a, when the laser scanning speed is low, the energy of the laser pulse accumulates on the material surface for a longer period of time, the fibers undergo more serious thermal damage, and the epoxy resin around the fibers is ablated and vaporized. When the laser scanning speed is increased, the heat accumulation on the surface of the material is smaller, and the isotherm distribution cloud in Figure 10b reveals that the heat along the laser scanning path is larger. When the laser scanning speed is 230 mm/s, the laser ablation damage is suppressed, and the heat-affected area of the laser is also relatively reduced, which is due to the decrease in the laser pulse overlap rate caused by the increase in the laser scanning speed, which reduces the width and depth of the laser ablation.

**Figure 11:**
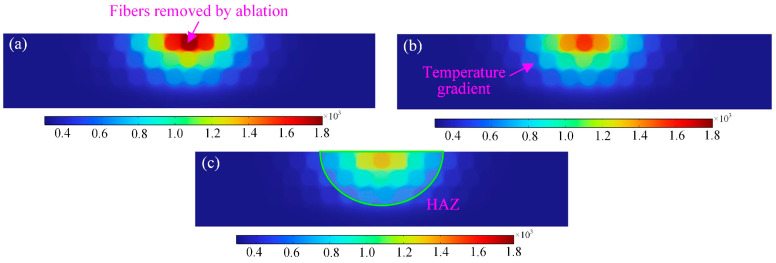
HAZ of cross-sections at different laser scanning speeds: (**a**) 30 mm/s, (**b**) 130 mm/s, and (**c**) 230 mm/s.

The variation in the cross-sectional heat-affected zone under different laser scanning speeds is demonstrated in Figure 11. As illustrated in Figure 11, the overall cross-sectional heat-affected zone of the material is semi-elliptical, and when the scanning speed is 30 mm/s, the material acts with the laser heat source for a long time, and more energy is transferred to the lower surface of the material, and at this time, the energy in the center of the laser irradiation is the highest. When the laser scanning speed increases, the temperature at the center of laser irradiation gradually decreases, the action time between the laser and the material decreases, and the heat absorbed by the material also decreases, but the temperature after laser irradiation is higher than the gasification threshold of the fibers, which leads to the formation of thermal damage morphology of the fibers in the center region by ablation. As shown in Figure 11c, the laser scanning speed is too fast, resulting in only a small number of carbon fibers being thermally affected, which ultimately leads to a reduction in the amount of material removed.

### 3.5. Effect of Laser Parameters on Groove Profile

**Figure 12:**
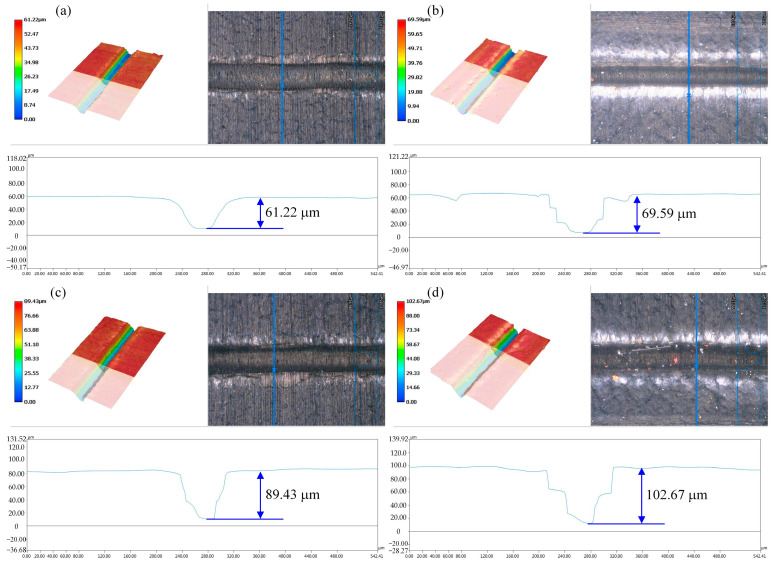
Contour variation in grooves at different laser powers: (**a**) 200 W, (**b**) 400 W, (**c**) 600 W, (**d**) 800 W.

Based on the above finite element simulation analysis, it can be found that the laser power, pulse frequency, and laser scanning speed need to be controlled to achieve high-quality and efficient processing of CFRP. From the point of view of processing efficiency, the laser power and scanning speed can be synergistically controlled. Therefore, the contour variation in laser single-scribing grooves at different laser powers was analyzed, as shown in Figure 12. As the laser power increases, there are obvious heat-affected zones on both sides of the grooves after laser scribing, and more material is removed. In addition, when processing with a nanosecond pulsed laser, there is heat accumulation between pulses. When the heat accumulation is higher than the pyrolysis temperature of the resin, the resin matrix around the grooves is removed. From Figure 12a,d, it can be found that the width and depth of the grooves after laser processing increased with the increase in the laser power. When the laser power increases from 200 W to 800 W, both the width and depth of the grooves increase rapidly, in which the depth also grows from 61.22 μm to 102.67 μm, and the grooves as a whole appear in an inverted triangle shape.

**Figure 13:**
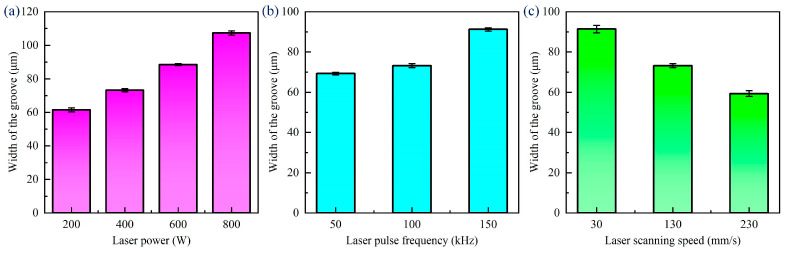
Variation patterns of width of grooves at different laser parameters: (**a**) laser power, (**b**) laser pulse frequency, (**c**) laser scanning speed.

Due to the specimen in the processing not being cleaned up before and after the processing, there is the presence of processing spatter and residue in the processing area. Splatter is mainly caused by the thermal effect of the processing process; in the processing process, the material will undergo the process of melting, boiling, and evaporation, the melting and boiling of the material will cause the splashing of the molten material, and the molten material will be cooled down after the formation of the splatter attached to the material surface. Therefore, before measuring the width and depth of the grooves after different laser scanning speeds, it is necessary to use an ultrasonic cleaner for surface treatment. Figure 13 shows the change rule of the width of the groove under different laser parameters. In the laser ablation experiments of CFRP grooves, we found that the width of the groove widened from 61.48 μm to 107.3 μm when the laser power was increased from 200 W to 800 W. The width of the groove increased from 69.28 μm to 91.24 μm when the laser pulse frequency was increased from 50 kHz to 150 kHz. On the contrary, the scanning speed of the laser was accelerated from 30 mm/s to 230 mm/s, and the width of the groove decreased from 91.43 μm to 59.32 μm. These data suggest that the increase in the laser power and frequency promotes more material removal, resulting in wider grooves, while the increase in the scanning speed reduces the laser–material interaction time, which in turn reduces the width of the groove.

**Figure 14:**
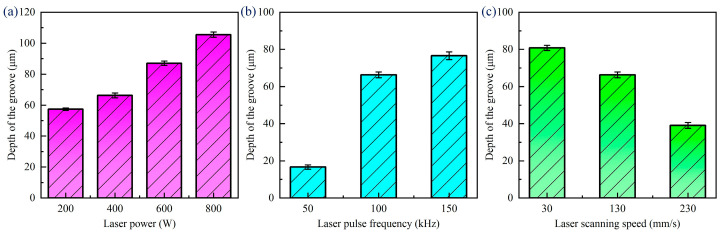
Variation patterns of depth of grooves at different laser parameters: (**a**) laser power, (**b**) laser pulse frequency, (**c**) laser scanning speed.

When laser ablating CFRP grooves, the laser power, pulse frequency, and scanning speed have a significant effect on the depth of the groove, as seen in Figure 14. The higher the laser power, the larger the groove width, because the high-power laser leads to more intense material ablation and vaporization. The greater the laser frequency, the greater the number of pulses acting on the material per unit time, the greater the cumulative energy, and the greater the depth of the groove accordingly. The higher the laser scanning speed, the lower the spot overlap, the lower the energy accumulation, resulting in a reduction in the depth of the groove. These laws indicate that the precise regulation of laser processing parameters is essential to achieve high-quality processing of CFRP grooves.

### 3.6. Effect of Laser Parameters on Surface Ablation Morphology

**Figure 15:**
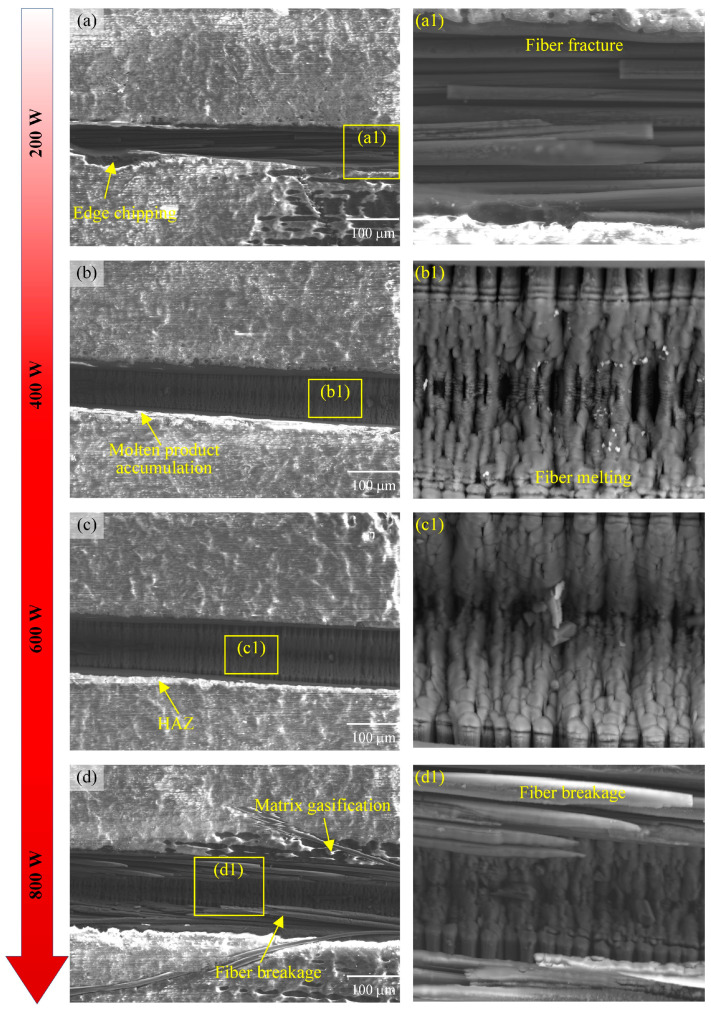
Surface morphology of grooves under different laser powers: (**a**) 200 W, (**b**) 400 W, (**c**) 600 W, (**d**) 800 W, and (**a1**)–(**d1**) are enlarged views of local areas.

The surface micromorphology of laser-ablated CFRP grooves shows significant features with the variation in the laser power, as shown in Figure 15. When the laser power was increased from 200 W to 400 W, the width of the grooves gradually increased and the edges were relatively neat, which was attributed to the increased material removal efficiency due to the enhanced laser energy. Upon further increase to 600 W, the groove width continues to expand, but ablation becomes apparent and slight melt product build-up and chipping begins to occur at the edges, due to more severe pyrolysis and cavitation of the material surface as a result of the widening of the heat-affected zone caused by the high laser energy. When the laser power reaches 800 W, the groove width increases significantly, but the ablation phenomenon is extremely serious, and a large amount of melt product build-up and obvious chipping appear at the edges, which is due to the rapid heating of the material caused by the excessively high laser energy and the generation of violent chemical reactions and mechanical exfoliation.

It can also be seen in Figure 4 that the surface was not completely removed at the lower laser power, and the fibers in the central region of the laser action were ablated. The fibers at the edges did not reach the ablation threshold and an oxidation reaction occurred. Then, when the laser power reached 800 W, the fiber ablation was severe, the resin vaporized to form a void, and the groove showed an inverted triangular shape as a whole. At the same time, there was an HAZ at the edge of the center of the laser action. In short, although the increase in the laser power can effectively increase the width of the groove, it also exacerbates the ablation phenomenon, leading to a decline in edge quality. The mechanism of laser power is mainly through the increase in energy density, to promote material removal and chemical reaction, but too high an energy will lead to unfavorable thermal effects and mechanical stripping.

**Figure 16:**
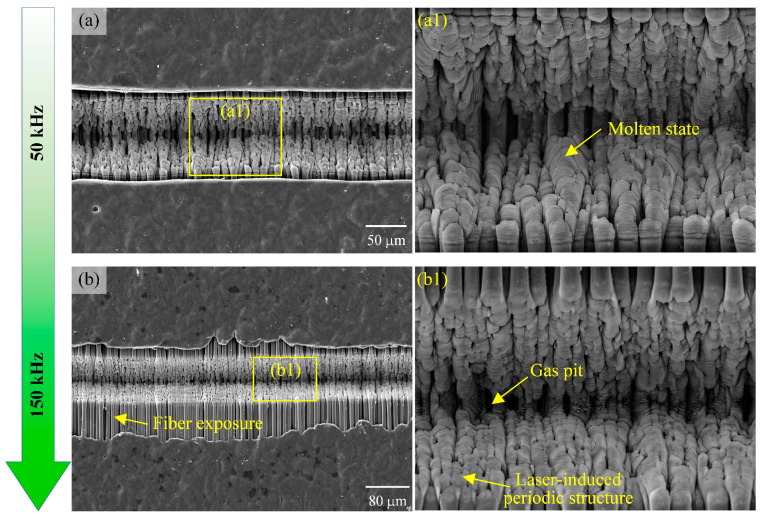
The effect of laser pulse frequency on the surface morphology of the grooves: (**a**) 50 kHz, (**b**) 150 kHz, and (**a1**,**b1**) are enlarged views of local areas.

The effect of the laser pulse frequency on the surface morphology of the grooves and the groove edges is shown in Figure 16. When the laser pulse frequency increased from 50 kHz to 150 kHz, the width of the grooves showed an increasing trend, but the ablation phenomenon also became more serious.

At a laser pulse frequency of 50 kHz, the fiber at the bottom of the grooves showed a molten state, forming a laser-induced periodic structure. At the same time, the substrate material undergoes hot gassing and vaporization, making the sides of the grooves relatively smooth. In this state, the thermal conductivity of the fiber is higher than that of the resin, resulting in rapid propagation of the laser energy through the fiber and thermal damage and vaporization of the surrounding resin, forming a specific heat-affected zone. When the laser pulse frequency was increased to 100 kHz, the ablation width of the grooves increased significantly and the fragmentation of the fibers became more pronounced. This is due to the fact that a higher frequency means more energy outputs in the same amount of time, which improves the processing efficiency but also increases the thermal damage to the material. A further increase in the laser pulse frequency to 150 kHz resulted in severe removal of the resin matrix, with chipping-like defects appearing at the edges of the grooves, and fibers in the unprocessed areas being exposed as a result. This result suggests that too high a laser frequency leads to excessive heat accumulation and excessive thermal damage to the material. As shown in Figure 6, the increase in the laser pulse frequency from 50 kHz to 150 kHz increases the energy density acting on the material per unit time, while helping to reduce the thermal accumulation effect. However, from the experimental results, it was found that the increase in the pulse frequency did increase the width and depth of the grooves, but too high a frequency also introduced an increase in surface roughness, which could be due to the concentration of thermal stresses caused by the overlap between pulses. The optimization of the pulse frequency can be considered later to make the best balance between machining quality and efficiency.

**Figure 17:**
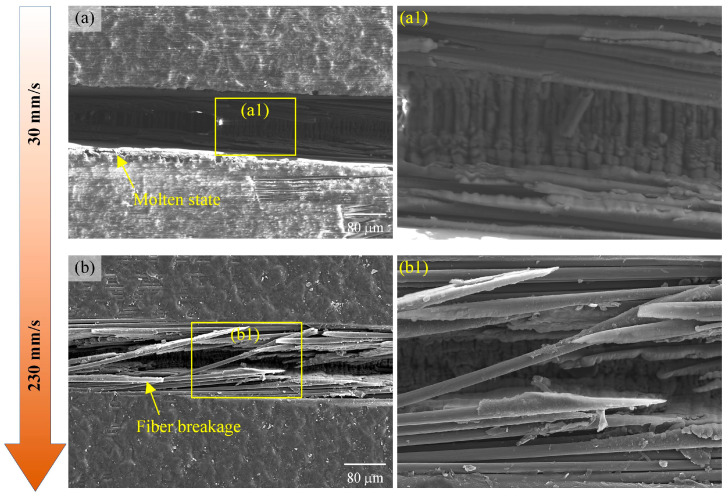
The effect of laser scanning speed on the surface morphology of the grooves: (**a**) 30 mm/s, (**b**) 230 mm/s, and (**a1**,**b1**) are enlarged views of local areas.

During laser ablation of CFRP grooves, the effect of the laser scanning speed on the surface morphology of the grooves is shown in Figure 17. When the laser scanning speed increases from 30 mm/s to 230 mm/s, the width of the groove decreases gradually. At the low scanning speed of 30 mm/s, the laser acts with the material for a long time and the energy accumulation is sufficient, resulting in a larger groove width and relatively smooth edges. However, as the scanning speed is increased to 230 mm/s, the groove width decreases and fiber fragmentation begins to appear, which is due to the accelerated scanning speed, and the laser energy is not sufficiently applied to the material, resulting in part of the area not being completely removed. At this time, there are a large number of unremoved fibers and matrixes, and the damage in the groove such as fiber fragmentation and fracture is more serious. This is because the excessive scanning speed leads to a very short residence time of the laser energy on the material surface, which is unable to effectively remove all the material, and at the same time, the mechanical stress generated by the high-speed scanning also exacerbates the damage of the fibers.

From comparison with the simulation results in Figure 8, it can be found that the accelerated scanning speed leads to a decrease in the laser–material interaction time, which reduces the material removal rate, but also helps to reduce the heat-affected zone and improve the machining accuracy. Figure 17 demonstrates that as the scanning speed increases, the material removal rate does decrease and the machined edges become less smooth, which is in general agreement with the simulation. In addition, too fast a scanning speed can also lead to insufficient energy density to effectively melt or vaporize the material, resulting in incomplete cutting or insufficient etching depth.

## 4. Conclusions

In this study, a finite element model considering the anisotropic heat transfer and non-homogeneity of CFRP was established to analyze the influence of laser parameters on processing quality. Combined with numerical simulations and experimental validations, the ablation mechanism, the evolution of the HAZ, and their dependence on the laser parameters were revealed. The key findings and their implications are summarized as follows. Firstly, the simulation results reveal that the thermal conductivity of CFRP differs significantly along the axial and radial directions of the carbon fibers, leading to an elliptical ablation morphology following laser irradiation. Notably, the nanosecond pulsed laser process demonstrates a thermal accumulation effect between pulses, which is influenced by the laser power and pulse frequency. This accumulation extends the cooling times at higher power and shorter pulse durations, which is critical for optimizing the processing efficiency. Secondly, the laser power, scanning speed, and pulse frequency have a pronounced impact on the width and depth of grooves during the single-pass laser grooving of CFRP by the simulations. Based on the experimental results, it was found that increased laser power leads to wider and deeper grooves and a larger HAZ, while a higher scanning speed reduces spot overlap, resulting in a smaller energy density, groove width, and depth. Next, excessive laser power or a low scanning speed causes severe ablation of the fibers, leading to significant thermal damage and HAZ expansion. Increasing the pulse frequency reduces the length of the temperature field along the laser scanning path while increasing its width perpendicular to the path. A higher pulse frequency results in greater energy density, raising the maximum surface temperature and enhancing energy transfer to deeper material layers, which intensifies ablation and HAZ formation. Finally, as the laser power increases or the scanning speed decreases, the grooves deepen and widen, adopting an inverted triangular shape. Heat accumulation between laser pulses becomes critical when the accumulated temperature exceeds the resin’s pyrolysis point, resulting in resin removal around the groove. The selection of the laser parameters significantly influences the ablation morphology, HAZ, and groove contour. Proper parameter optimization is essential to prevent excessive heat accumulation and over-ablation, ensuring high machining accuracy and efficiency.

This research provides a comprehensive understanding of the laser–material interaction mechanisms in CFRP machining, offering quantitative insights into the effects of laser parameters. The findings guide parameter selection for nanosecond pulsed laser processing to minimize thermal damage and improve machining precision. This study not only advances the theoretical foundation of CFRP laser machining, but also lays a solid foundation for the broader adoption of laser technologies in composite material manufacturing.

## Figures and Tables

**Table 1:** Specific physical parameters of CFRP [42,43,44,45,46].

Name	Unit	Value
Atomic mass of fiber	kg	1.993 × 10^−26^
Density of fiber	kg/m^3^	1850
Density of epoxy resin	kg/m^3^	1250
Thermal diffusivity of fiber	cm^2^·s^−1^	0.38
Thermal diffusivity of epoxy resin	cm^2^·s^−1^	0.001
Thermal conductivity of fiber	W/m/K	5
Thermal conductivity of epoxy resin	W/m/K	0.2
Specific heat capacity of fiber	J/kg/K	710
Specific heat capacity of epoxy resin	J/kg/K	1200
Vaporization temperature of fiber	°C	3627
Vaporization temperature of epoxy resin	°C	527
Decomposition temperature of fiber	°C	880
Decomposition temperature of epoxy resin	°C	425

**Table 2:** Specific parameters for nanosecond laser simulation.

Name	Unit	Value
Scanning speed	mm/s	30/130/230
Power	W	200/400/600/800
Pulse repetition rate	kHz	50/100/150
Spot diameter	μm	35
Pulse width	ns	16.8
Laser wavelength	nm	355

## Data Availability

The original contributions presented in the study are included in the article, further inquiries can be directed to the corresponding authors.
